# Red Meat Hypersensitivity and Probable Alpha-Gal Syndrome: Prevalence Among Adolescents

**DOI:** 10.7759/cureus.55403

**Published:** 2024-03-02

**Authors:** Martín Bedolla-Barajas, Jaime Morales-Romero, Carlos Meza-Lopez, Tonatiuh R Bedolla-Pulido, Wilbert Mendoza-Solís, Fernanda Novelo-del Muro, Diana I Juárez-Luna

**Affiliations:** 1 Allergy and Clinical Immunology, Nuevo Hospital Civil de Guadalajara “Dr. Juan I. Menchaca”, Guadalajara, MEX; 2 Public Health, Universidad Veracruzana, Xalapa, MEX; 3 Pediatrics and Child Health, Nuevo Hospital Civil de Guadalajara “Dr. Juan I. Menchaca”, Guadalajara, MEX; 4 Pediatrics and Neonatology, Nuevo Hospital Civil de Guadalajara “Dr. Juan I. Menchaca”, Guadalajara, MEX; 5 Medicine, Universidad Guadalajara Lamar, Guadalajara, MEX; 6 Medicine, Univesidad de Guadalajara, Centro Universitario de Ciencia de la Salud, Guadalajara, MEX

**Keywords:** adolescence, prevalence, alpha-gal syndrome, alpha-gal allergy, red meat allergy

## Abstract

Introduction. It is unknown whether late adolescents represent a particular risk group for the development of red meat hypersensitivity (RMH) and alpha-gal syndrome (AGS). This age group's physiological changes and eating habits could play a determining role. This study aimed to estimate the self-reported prevalence of RMH and probable AGS among late adolescents.

Methods. A cross-sectional study analyzed a sample of 1992 Mexican adolescents between 15 and 18 years of age. The data were obtained with a previously validated questionnaire that asked about the clinical manifestations related to red meat intake. Confidence intervals at 95% (95% CI) were estimated for proportions.

Results. In total, there were 19 adolescents with RMH, a prevalence of 1.0% (95% CI: 0.6-1.5%). The main red meats related to symptoms were pork (89.5%), beef (21.1%), lamb (10.5%), and mutton (5.1%). The most frequent manifestations of RMH were gastrointestinal (73.7%), respiratory (63.2%), and cutaneous (63.2%). Once the symptoms were grouped, there were two cases of urticaria (2/19, 10.5%) and six cases with probable anaphylaxis (6/19, 31.6%). Finally, three adolescents were considered probable cases of AGS, which represents a prevalence of 0.15% (95% CI: 0.1-0.4%).

Conclusion. Although the prevalence of RMH in late adolescents is low, early detection is justified because approximately one-third present with severe symptoms.

## Introduction

International organizations recommend implementing sustainable, healthy diets with the aim of promoting people's well-being. These diets include the consumption of cereals, nuts, fruits, vegetables, poultry, fish, and small portions of red meat [1]. Traditionally, the Mexican diet is composed of foods that belong to the following groups: grains, tubers, legumes, and vegetables. Specifically, the diet includes foods such as corn, beans, chili, pumpkin, tomato, and onion, and less frequently, it includes products derived from corn, fruits, beverages, fish and shellfish, meats, sweets, and sweeteners, as well as herbs and condiments [2]. In the meat production industry, dominated by China and the United States, Mexico is positioned as the sixth most important country worldwide; on the other hand, when evaluating the per capita consumption of meat, the list is headed by the United States, followed by Argentina, Brazil, and the European Union; in this sense, Mexico is positioned in fifth place [3]. However, in Mexico, adolescents prefer to consume foods that are not recommended for daily consumption, such as sweetened drinks, sweets, and desserts, as well as sweetened cereals; in smaller quantities, they consume recommended foods such as unprocessed meats, dairy products, and nuts [4].

The prevalence of hypersensitivity to foods is clearly influenced by their availability as well as by the eating habits and cultural context of each population; thus, in Canada, the prevalence among children is 2.5%, while in the United States, this figure rises to 3.4%; among the main foods responsible for producing symptoms are peanuts, shellfish, eggs, or milk [5,6]. In various European countries, the prevalence ranges from 1.9% to 5.6%; in those same countries, milk, celery, and apples are the foods responsible for the discomfort [7]. On the other hand, in China, up to 6.2% of children have symptoms after consuming food, mainly shrimp, mango, and mollusks [8]. Notably, in previous studies, red meat was not listed among the leading causes of food allergies in children. Red meat is considered to be derived from sheep, cattle, and pigs, which is a source of essential nutrients [9], and its high consumption has been pointed out as a risk factor for chronic degenerative diseases such as cancer [10]. However, little has been analyzed about the role of its consumption in the production of allergies. In this regard, there is a picture of a food allergy derived from the consumption of red meat that has been called alpha-gal syndrome (AGS). Its name derives from the galactose-alpha-1,3-galactose molecule inoculated by the bite of a certain type of tick; therefore, in this event, the subject can develop potentially serious allergic reactions after eating red meat [11]. AGS has been widely described in the United States, Australia, Europe, and Japan [12], but little is known about its prevalence in Latin America. Food allergies used to be considered a problem that mainly affected children in their first years of life. However, currently, an increase in the prevalence of food allergies among adolescents and adults has been observed. Physiological changes and eating habits in late adolescents could play a determining role in developing red meat hypersensitivity (RMH) and AGS. The prevalence of this group is unknown. Thus, the main objective of this study was to estimate the prevalence of RMH and to explore the existence of probable cases of AGS in a sample of late adolescents.

## Materials and methods

Design

This research corresponds to a secondary analysis, with a cross-sectional design, of the survey applied by Bedolla-Barajas to a representative sample of Mexican adolescents, from 15 to 18 years old, residents of Guadalajara, Mexico.

Sampling

The study population was divided into strata so that the prevalences were not influenced by any sociodemographic characteristic (possible confounding factor). The sampling applied from April to June 2016 was probabilistic, stratified, and clustered. The initial sampling frame consisted of high schools. For this study, Guadalajara was operationally divided into seven strata, from which at least one school was randomly selected (a cluster) from each. Within each selected school, a random sample was again used, considering school grades as new strata. Using proportional allocation, the final sample size of selected adolescents was n=1992. A more detailed description of this sampling can be found in the previously published works mentioned above [13,14].

Instrument

A structured questionnaire previously validated in Mexico was applied, which included the adolescent's age, sex, personal history of atopic and non-atopic diseases, and family history of allergic diseases. In the validation process, an agreement of 0.77 was obtained, according to the Kappa index, and a Cronbach's alpha of 0.86 [15]. The prevalence of asthma, allergic rhinitis, atopic dermatitis, and food allergies derived from this survey was previously published [13,14].

Definitions

The following operational definitions were adapted for this study: In principle, food hypersensitivity was determined by the question: Do you suffer from allergic reactions after eating food or drinking beverages? [15]. If they answered affirmatively, they were asked whether or not the symptoms were associated with eating red meat. RMH was defined as a condition encompassing all cases in which individuals report discomfort after eating red meat, regardless of their medical history or the presence of confirmatory tests [16]. Oral allergy syndrome refers to the presence of oral symptoms, such as itching or swelling in the mouth or throat, after consuming red meat specifically. Probable anaphylaxis was considered when the affected organs and the symptoms were characteristic of allergic reactions and affected two or more organs [17]. Finally, a probable AGS was determined when the symptoms of urticaria or anaphylaxis manifested 2 hours or more after eating some type of red meat.

Statistical analysis

The prevalence of RMH and probable AGS was obtained by dividing the number of adolescents with symptoms after eating red meat (according to the aforementioned definitions) by the total number of participants. Additionally, 95% Wilson confidence intervals (score) were estimated for proportions. In the comparison of qualitative variables, the chi-square test or Fisher's exact test was used as necessary. Statistical significance was set at a p<0.05. SPSS Statistics version 20.0 (IBM Corp. Released 2011. IBM SPSS Statistics for Windows, Version 20.0. Armonk, NY: IBM Corp.) was used to perform the statistical analyses. 

Ethics

This research complied with the ethical principles established in the Helsinki Declaration proposed by the World Medical Assembly for medical research in human beings and was approved by the Ethics and Research Committee of Nuevo Hospital Civil de Guadalajara “Dr. Juan I. Menchaca” (approval number: CEI-00066). Before carrying out the surveys, the teachers at the participating schools were presented with the objective of the study, and their collaboration was requested to survey the students. Once their approval was obtained, the students were provided with detailed information about the research, and if they agreed, they gave their verbal consent to be included in the study.

## Results

This study included 1,056 women (53%), and 936 men (47%). A total of 19 adolescents with RMH were identified, with a prevalence of 1.0% (95% CI: 0.6-1.5%). It was observed that atopic comorbidity (asthma, allergic rhinitis, or atopic dermatitis) was significantly more frequent in adolescents with RMH compared to those who did not report complaints after consumption of red meat (p<0.05). On the other hand, the frequency of family history of asthma or allergic rhinitis was higher in the group with RMH (p<0.05) (Table 1).

**Table 1:** Population characteristics Comparison of proportions using the chi-square test or Fisher's exact test when necessary RMH: red meat hypersensitivity

	RMH				
	Yes		No		
	N=19		N=1973		
Variable	n	%	n	%	p-value
Sex female	14	73.7	1,042	52.8	0.070
Atopic comorbidity					
Asthma	7	36.8	245	12.4	0.001
Allergic rhinitis	7	36.8	173	8.8	<0.0001
Atopic dermatitis	4	21.1	99	5.0	0.002
Family history of atopic diseases					
Asthma	10	52.6	442	22.4	0.002
Allergic rhinitis	5	26.3	207	10.5	0.044
Atopic dermatitis	1	5.3	100	5.1	0.999
Current tobacco consumption	0	0	204	10.3	0.139

The main red meats related to symptoms were pork (89.5%), beef (21.1%), lamb (10.5%), and mutton (5.1%). The most frequent symptoms of RMH were gastrointestinal (73.7%), followed by respiratory (63.2%) and cutaneous (63.2%).

Among the intestinal complaints, abdominal pain and heartburn stood out, while among the respiratory complaints were respiratory distress and sneezing, followed by nasal congestion. Regarding skin complaints, reddish skin and itching on the face were the most frequent symptoms (Table 2).

**Table 2:** Clinical manifestations after consumption of red meat Comparison of proportions using the chi-square test or Fisher's exact test when necessary RMH: red meat hypersensitivity

	RMH				
	Yes		No		
	N=19		N=1973		
Symptoms	n	%	n	%	p-value
Gastrointestinal symptoms					
Abdominal pain	7	36.8	38	1.9	<0.0001
Heartburn	7	36.8	34	1.7	<0.0001
Itchy lips	4	21.1	55	2.8	0.002
Lip swelling	4	21.1	47	2.4	0.001
Vomiting	4	21.1	31	1.6	<0.0001
Bloating	3	15.8	24	1.2	0.002
Flatulence	2	10.5	24	1.2	0.024
Diarrhea	1	5.3	35	1.8	0.294
Respiratory symptoms					
Shortness of breath	9	47.4	42	2.1	<0.0001
Sneezing	8	42.1	42	2.1	<0.0001
Nasal congestion	6	31.6	33	1.7	<0.0001
Rhinorrhea	5	26.3	32	1.6	<0.0001
Cough	5	26.3	31	1.6	<0.0001
Throat tightness	4	21.1	60	3.0	0.003
Wheezing	2	10.5	18	0.9	0.015
Cutaneous symptoms					
Reddish skin	10	52.6	61	3.1	<0.0001
Itchy face	7	36.8	30	1.5	<0.0001
Hives	5	26.3	39	2.0	<0.0001
Body itching	3	15.8	41	2.1	0.008

After grouping the symptoms, it was observed that one adolescent presented with oral allergy syndrome (1/19, 5.3%), while urticaria occurred in two cases (2/19, 10.5%) and six cases of probable anaphylaxis (6/19, 31.6%). In the remaining cases, the symptoms were more of a non-specific nature. Of the total cases with RMH, three cases were compatible with AGS, which represents an overall prevalence of 0.15% (95% CI: 0.1-0.4%); two of them were men and one woman; in all three cases, the consumption of pork was responsible. Two of them manifested urticaria, and one more was a probable anaphylactic reaction (Table 3).

**Table 3:** Clinical characteristics of adolescents with RMH F: female, M: male, AR: allergic rhinitis, AD: atopic dermatitis, OAS: oral allergy syndrome, RMH: red meat hypersensitivity *Time in minutes

Case	Sex	Comorbidity			Pollen allergy	Symptom onset time*	Type of red meat				Symptoms		
		Asthma	AR	AD			Pork	Beef	Lamb	Mutton	OAS	Urticaria	Probable anaphylaxis
1	F	-	-	-	-	<120	+	+	-	-	-	-	-
2	M	-	-	-	-	>120	+	-	-	-	-	+	-
3	F	-	+	-	-	<120	+	-	+	+	-	-	-
4	F	-	+	-	-	<120	+	-	-	-	-	-	-
5	F	-	-	-	-	>120	-	+	-	-	-	-	-
6	F	-	-	+	-	<120	+	-	-	-	-	-	-
7	M	-	-	-	-	>120	+	-	-	-	-	+	-
8	F	+	-	-	-	<120	+	-	-	-	-	-	+
9	F	-	-	+	-	<120	-	+	-	-	-	-	-
10	F	+	+	+	+	>120	+	-	-	-	-	-	+
11	F	-	+	-	+	<120	+	-	-	-	+	-	-
12	F	+	+	-	+	<120	+	-	-	-	-	-	+
13	M	+	-	-	+	<120	+	-	-	-	-	-	+
14	M	-	-	-	+	<120	+	-	-	-	-	-	-
15	F	+	-	-	+	>120	+	-	-	-	-	-	-
16	M	+	-	+	+	<120	+	+	-	-	-	-	-
17	F	+	+	-	+	<120	+	-	-	-	-	-	+
18	F	-	-	-	+	<120	+	-	-	-	-	-	-
19	F	-	+	-	+	<120	+	-	+	-	-	-	+

## Discussion

Prevalences of RMH and AGS

In our study, we observed that the prevalence of RMH among late Mexican adolescents is 1.0%. We also observed that it was pork meat that was mainly associated with clinical symptoms (89.5%), from which intestinal (73.7%) and respiratory (63.2%) complaints stood out. To the best of our knowledge, it is relevant to highlight that this is one of the first population-based studies in Latin America that describes at least three possible cases of AGS in the group of adolescents with RMH (0.15%).

In this study, it was observed that a minority of the late adolescents analyzed experienced some type of discomfort after having consumed red meat (1%). Among them, nine cases were identified with symptoms compatible with a probable allergic reaction to red meat (oral allergy syndrome, urticaria, and anaphylaxis), which represents a prevalence of 0.45% (95% CI: 0.22-0.87%).

In Asia, various studies in children and adults have shown that the frequency of RMH is low, similar to what we observed in our study. For example, in China, two previous studies showed that the frequency of beef allergy among schoolchildren was 0.3% to 0.5% [8,18], while allergy to mutton was 0.4% [8]. In other countries in the same region, similar findings have been observed. In Hong Kong, data from more than 7,000 children and adolescents was analyzed to determine the prevalence of food hypersensitivity, which was found to be 4.8%. Red meats accounted for only 15 cases, resulting in an overall frequency of 0.2% [19]. The prevalence of RMH in nearly 24,000 children attending daycare centers in Japan was 0.2% [20]. Finally, in Korea, the prevalence of RMH in adolescents aged 12 to 13 years was as follows: pork 0.1% and beef 0.07%. Among the 15-16-year-old group, it was 0.17% for pork and 0.04% for beef [21]. On the other hand, through the Canadian Primary Care Sentinel Surveillance Network, data corresponding to 288,490 children in Canada were analyzed, and it was observed that the prevalence of food allergies reported by physicians was 2.5%; however, red meat was not identified as a major cause of symptoms in children in this study. In the Europrevall study, which analyzed the frequency of food sensitization among children between seven and 10 years of age from seven cities in Europe, no evidence of allergy to red meat was found [7], as was a study carried out in Canada [5] and another in the United States [6]. In our country, a study was carried out with a selected sample of the pediatric population who attended consultations with allergists. In this study, 127 cases of allergy associated with the intake of red meat were identified among a total of 1971 patients, a prevalence of allergy to red meat of 6.4% [22]. The late introduction of cattle, sheep, goats, pigs, and seafood as food sources in our country is a significant factor that must be considered when interpreting our results. It seems that this event is acting as a predisposing factor for the high frequency of adverse reactions associated with the consumption of red meat compared to other countries. In summary, our results, in conjunction with those previously shown, highlight that red meat allergy varies geographically and highlight the importance of further research to better understand this occurrence.

Possible implications of the results

The prevalence of AGS in the open population has rarely been analyzed; this study is one of the first to explore this possibility with a sample of late adolescents. According to the results, the prevalence was 0.15%. Most studies that have determined the prevalence of AGS have used the frequency of IgE-mediated sensitization against alpha-gal as a starting point. According to a population-based cross-sectional study conducted in Denmark and Spain, 5.5% and 8.1% of adults, respectively, were found to have positive serum IgE against alpha-gal [23]. On the other hand, in populations with a higher risk of being bitten by ticks, the frequency of allergic sensitization is higher. In Spain, it was observed that workers in the forests had a frequency of allergic sensitization to alpha-gal of 15%, compared to the control group, which was 4% [24]. In Germany, 35% of forest workers and hunters were sensitized to alpha-gal, compared to 15% of the control group [25]. Additionally, in the United States, timber harvesters and forestry and wildlife professionals had a 40% frequency of allergic sensitization [26]. A recent multicenter study in children revealed that the prevalence of serum IgE against alpha-gal was 32% in Ecuador, 54% in Kenya, and 25% in Virginia (United States) [12]. Furthermore, AGS may be closely associated with tick bites, but there is also the possibility that it is linked to exposure to other sources of alpha-gal, such as certain drugs or gelatin; more population-based studies are required to determine its prevalence more precisely. Finally, it should be highlighted that the majority of AGS cases were initially located in the United States, particularly in the southeastern region. An increasing number of AGS cases are now being reported in different parts of the world. As we mentioned, there could also be cases of AGS-like reactions, not necessarily AGS, among adolescents with RMH.

Notably, both in the RMH group and in the AGS group, almost a third of each of them experienced symptoms compatible with an anaphylactic reaction. Similar findings were observed in the United States, where 60% of 261 adult subjects with allergic sensitization to alpha-gal had anaphylaxis as a manifestation of AGS [27]. In Europe, a study with 128 patients showed that 47% of them experienced anaphylaxis after consuming this type of meat [28].

In addition to AGS, cat-pig syndrome is another possibility to consider among the 19 adolescents with RMH. It was observed that five adolescents experienced discomfort two hours or more after consuming red meat, and it was identified that three of them were probable cases of AGS; thus, it is probable that some of the 14 remaining cases could correspond to cases of cat-pig syndrome. In this syndrome, patients produce specific IgE against cat serum albumin, which cross-reacts with porcine albumin. The syndrome is characterized by severe and, in some cases, fatal allergic reactions; unlike AGS, the onset of symptoms is immediately after consumption of pork and is usually accompanied by oral pruritus [29].

Limitations and strengths of the study

It is important to consider that the data were obtained through a self-reported survey, which could have introduced an information bias based on subjectivity in the perception of the symptoms reported by adolescents. Regarding the AGS, this study has the limitation that it only focused on adolescents who lived within an industrialized city, which limits the generalization of the results to those who live in rural areas where it is expected that there will be greater exposure to ticks, which could influence the prevalence of RMH. Another limitation is related to how red meat is prepared and consumed. It's possible that the use of seasonings or other types of additives during the meat preparation may be responsible for the symptoms rather than the red meat itself. On the other hand, in cases with a high suspicion of AGS, the history of having suffered tick bites was not considered because this question was not part of the original survey; neither was there a clinical history nor a test to detect allergic sensitization to alpha-gal. Regarding the latter, it is important to mention that contact with other secondary or tertiary sources of alpha-gal could also have contributed to the process of allergic sensitization and the presence of symptoms associated with the consumption of red meat [30].

In our study, three possible cases of AGS were identified among a total of 1992 adolescents, two of whom experienced urticaria and one presented symptoms of anaphylaxis after having consumed pork. However, it is important to note that, due to the study design, the participants' history of tick bites was not specifically inquired about. An additional mention should be made regarding the sample size, which was initially calculated in a previous study by our working group, where the sample size was originally n=1981 (n=1992 subjects were finally recruited) [13], considering an estimated prevalence of food hypersensitivity of 50%, a confidence level of 95%, and a margin of error of ±2.3%. However, if we consider the prevalence of RMH reported in this study, which was 1%, then the sample size supports a confidence level of 99.9% (an error margin of 1%). The above increases the precision of our results.

## Conclusions

We found that the consumption of red meat, especially pork, is related to various symptoms in adolescents with RMH. Furthermore, having detected possible cases of AGS, a rare allergy that can be life-threatening and is related to the consumption of red meat, suggests the existence of some species of ticks in our country that could be associated with AGS. More studies are required in this regard.

## References

[1] (2020). Sustainable healthy diets: guiding principles (Article in Spanish). https://www.fao.org/3/ca6640es/CA6640ES.pdf..

[2] Valerino-Perea S, Lara-Castor L, Armstrong ME, Papadaki A (2019). Definition of the traditional Mexican diet and its role in health: a systematic review. Nutrients.

[3] (2022). Statistics Compendium 2022. Compendio estadístico.

[4] Gaona-Pineda EB, Rodríguez-Ramírez S, Medina-Zacarías MC, Valenzuela-Bravo DG, Martinez-Tapia B, Arango-Angarita A (2023). Consumers of food groups in the Mexican population. Ensanut Continuous 2020-2022 (Article in Spanish). Salud Publica Mex.

[5] Singer AG, Kosowan L, Soller L, Chan ES, Nankissoor NN, Phung RR, Abrams EM (2021). Prevalence of physician-reported food allergy in Canadian children. J Allergy Clin Immunol Pract.

[6] Tseng H, Vastardi MA (2023). Food insecurity and food allergy in children: a cross-sectional study. J Allergy Clin Immunol Pract.

[7] Lyons SA, Clausen M, Knulst AC (2020). Prevalence of food sensitization and food allergy in children across Europe. J Allergy Clin Immunol Pract.

[8] Feng H, Luo N, Lu Y (2022). Prevalence of parent-reported food allergy among children in China: a population-based cross-sectional survey. Front Immunol.

[9] McAfee AJ, McSorley EM, Cuskelly GJ, Moss BW, Wallace JM, Bonham MP, Fearon AM (2010). Red meat consumption: an overview of the risks and benefits. Meat Sci.

[10] Farvid MS, Sidahmed E, Spence ND, Mante Angua K, Rosner BA, Barnett JB (2021). Consumption of red meat and processed meat and cancer incidence: a systematic review and meta-analysis of prospective studies. Eur J Epidemiol.

[11] Young I, Prematunge C, Pussegoda K, Corrin T, Waddell L (2021). Tick exposures and alpha-gal syndrome: a systematic review of the evidence. Ticks Tick Borne Dis.

[12] Wilson JM, Keshavarz B, James HR (2021). α-Gal specific-IgE prevalence and levels in Ecuador and Kenya: relation to diet, parasites, and IgG(4). J Allergy Clin Immunol.

[13] Bedolla-Pulido TR, Bedolla-Barajas M, Morales-Romero J, Bedolla-Pulido TI, Domínguez-García MV, Hernández-Colín DD, Flores-Merino MV (2019). Self-reported hypersensitivity and allergy to foods amongst Mexican adolescents: prevalence and associated factors. Allergol Immunopathol (Madr).

[14] Morales-Romero J, Bedolla-Pulido TI, Bedolla-Pulido TR (2020). Asthma prevalence, but not allergic rhinitis nor atopic dermatitis, is associated to exposure to dogs in adolescents. Allergol Immunopathol (Madr).

[15] Puente-Fernández C, Maya-Hernández RL, Flores-Merino MV, Romero-Figueroa Mdel S, Bedolla-Barajas M, Domínguez García MV (2016). Self-reported prevalence and risk factors associated with food hypersensitivity in Mexican young adults. Ann Allergy Asthma Immunol.

[16] Ben-Shoshan M, Harrington DW, Soller L (2010). A population-based study on peanut, tree nut, fish, shellfish, and sesame allergy prevalence in Canada. J Allergy Clin Immunol.

[17] Sicherer SH, Munoz-Furlong A, Burks AW, Sampson HA (1999). Prevalence of peanut and tree nut allergy in the US determined by a random digit dial telephone survey. J Allergy Clin Immunol.

[18] Feng H, Luo N, Chen F (2022). Self-reported food allergy prevalence among elementary school children - Nanchang City, Jiangxi Province, China, 2021. China CDC Wkly.

[19] Ho MH, Lee SL, Wong WH, Ip P, Lau YL (2012). revalence of self-reported food allergy in Hong Kong children and teens--a population survey. Asian Pac J Allergy Immunol.

[20] Kaneko M, Miyoshi T, Miyashita Y (2021). Food allergy in nursery children of Kawasaki city, Japan. Asian Pac J Allergy Immunol.

[21] Kim M, Lee JY, Jeon HY (2017). Prevalence of immediate-type food allergy in Korean schoolchildren in 2015: a nationwide, population-based study. Allergy Asthma Immunol Res.

[22] Medina-Hernandez A, Huerta-Hernandez RE, Gongora-Melendez MA (2015). Clinical-epidemiological profile of patients with suspicion of alimentary allergy in Mexico. Mexipreval Study (Article in Spanish). Rev Alerg Mex.

[23] Gonzalez-Quintela A, Dam Laursen AS, Vidal C, Skaaby T, Gude F, Linneberg A (2014). IgE antibodies to alpha-gal in the general adult population: relationship with tick bites, atopy, and cat ownership. Clin Exp Allergy.

[24] Venturini M, Lobera T, Sebastián A, Portillo A, Oteo JA (2018). Ige to α-Gal in foresters and forest workers from La Rioja, north of Spain. J Investig Allergol Clin Immunol.

[25] Fischer J, Lupberger E, Hebsaker J (2017). Prevalence of type I sensitization to alpha-gal in forest service employees and hunters. Allergy.

[26] Bellamy P, Sanderson WT, Winter K, Stringer JW, Kussainov N, Commins SP (2021). Prevalence of alpha-gal sensitization among Kentucky timber harvesters and forestry and wildlife practitioners. J Allergy Clin Immunol Pract.

[27] Wilson JM, Schuyler AJ, Workman L (2019). Investigation into the α-gal syndrome: characteristics of 261 children and adults reporting red meat allergy. J Allergy Clin Immunol Pract.

[28] Kiewiet MB, Apostolovic D, Starkhammar M, Grundström J, Hamsten C, van Hage M (2020). Clinical and serological characterization of the α-gal syndrome-importance of atopy for symptom severity in a European cohort. J Allergy Clin Immunol Pract.

[29] Posthumus J, James HR, Lane CJ, Matos LA, Platts-Mills TA, Commins SP (2013). Initial description of pork-cat syndrome in the United States. J Allergy Clin Immunol.

[30] Platts-Mills TA, Li RC, Keshavarz B, Smith AR, Wilson JM (2020). Diagnosis and management of patients with the α-gal syndrome. J Allergy Clin Immunol Pract.

